# Clinical outcomes of two-stage revision arthroplasty using a spiked tibial cement spacer in infected total knee arthroplasty

**DOI:** 10.1007/s00264-024-06257-7

**Published:** 2024-07-27

**Authors:** Ki-Bong Park, Jong-Min Kim, Bum-Sik Lee, Min-Seok Kim, Jae-Hwan Park

**Affiliations:** 1 Department of Orthopedic Surgery, Ulsan University Hospital, University of Ulsan College of Medicine, Ulsan, Republic of Korea; 2 Department of Orthopedic Surgery, Asan Medical Center, University of Ulsan College of Medicine, 288-1, Poongnap-dong, Songpa-gu, Seoul, 137-736 Republic of Korea

**Keywords:** Infection, Total knee arthroplasty, Revision, Spiked cement spacer, Function, Recurrence

## Abstract

**Purpose:**

A tibial cement spacer (TCS) with spikes offers better initial stability than a conventional TCS and reduces spacer-related problems in two-stage revision total knee arthroplasty (R-TKA) for infection. We compared the clinical outcomes of two-stage revision arthroplasty for infected TKA using spiked TCS with that of conventional TCS.

**Methods:**

This retrospective cohort study included 29 patients who underwent two-stage revision arthroplasty using an articulating cement spacer and who could be followed up for at least one year. Group S comprised 14 patients using spiked TCS, whereas Group C comprised 15 patients using conventional TCS. Demographic data, the interval from first to second stage revision, motion arc, numerical rating scale (NRS), Knee Society (KS) score, serum levels of erythrocyte sedimentation rate (ESR) and C-reactive protein (CRP), and frequency of repeating the first-stage and infection recurrence after R-TKA between the groups were analyzed.

**Results:**

No significant differences were observed in the female ratio and mean age between both groups. The mean interval between the first and second stage revision was significantly shorter in Group S than in Group C. The mean motion arc was significantly larger in Group S than in Group C. The mean NRS was significantly lower in Group S than in Group C. The mean KS score in Group S was significantly higher than that in Group C. Serum ESR and CRP levels did not differ between the groups. The frequency of repeating the first stage was lower in Group S than in Group C. However, the recurrence rate after R-TKA was higher in Group S than in Group C.

**Conclusion:**

Compared with conventional TCS, spiked TCS shortened the period until R-TKA and improved pain and function levels. However, no significant difference existed in the rate of infection recurrence after R-TKA.

## Introduction

Revision of infected total knee arthroplasty (TKA) is technically more difficult, has greater haemodynamic variations, and produces inferior surgical outcomes compared to primary TKA [1–4].

Typically, two-stage revision arthroplasty, in which the infected prosthesis is removed, a spacer containing cement is temporarily inserted, and then revision arthroplasty is performed after a complete cure of the infection, is a well-tolerated and effective procedure with a high success rate [5–7]. However, various problems related to cement spacers, including bone loss, cement spacer translation, cement spacer fracture, knee dislocation, and periprosthetic fracture, have been reported after performing first-stage procedures [8, 9].

Previous studies of a modified articulating cement spacer (ACS) technique that slightly manipulates the cement spacer to address these problems have revealed satisfactory results, with a zero-percentage prevalence of spacer-related problems [10–12].

The spiked tibial cement spacer (TCS) technique, a modified ACS technique, has been reported to improve initial stability compared to conventional TCS [12]. The clinical results of two-stage revision arthroplasty using spiked TCS have not yet been reported. Research on the effectiveness of spiked TCS for facilitating joint mobility and eradicating infection after the revision TKA (R-TKA) is essential for spiked TCS to become widely used in clinical practice.

We hypothesized that spiked TCS would be more effective than conventional TCS in two-stage revision arthroplasty for infected TKA. To evaluate this hypothesis, this study compared the increase in ultimate joint mobility and reduction in infection recurrence in patients diagnosed with infected TKA who underwent two-stage revision arthroplasty when spiked TCS was used.

## Materials and methods

### Study design and population

This retrospective case-control study collected clinical data from patients who were treated for infected TKA or infected R-TKA between August 2019 and November 2022. Infection was diagnosed based on white blood cell (WBC) count, erythrocyte sedimentation rate (ESR), C-reactive protein (CRP) levels, radiographic findings, and bacterial culture results. In total, 29 patients (29 knees) who underwent two-stage revision arthroplasty using an ACS were included in this study. Four patients underwent primary TKA at our institution (by a senior surgeon), and the remaining 25 patients were transferred from other institutions after being diagnosed with infected TKA or R-TKA. There were 25 women and four men with a mean age of 74.2 years at the time of the first-stage procedure. Causative microorganisms were isolated from 22 knees; the causative agent of infection was unknown in seven knees (24.1%). Methicillin-sensitive *Staphylococcus aureus* (24.1%), *Escherichia coli* (20.7%), methicillin-resistant *Staphylococcus aureus* (13.8%), *Staphylococcus agalactiae* (10.3%), and *Staphylococcus epidermidis* (6.9%) were the most common infecting organisms. The mean follow-up period following R-TKA was 26.1 months (range, 13.4–54.0 months) after surgery. Table 1 presents the characteristics of the two study populations.

**Table 1:** Clinical characteristics of 29 patients with infected total knee arthroplasty who underwent two-stage revision arthroplasty

	Group S (*n* = 14)	Group C (*n* = 15)	*P* value
Female	12 (85.7%)	13 (86.7%)	0.94
Age, years			
At primary TKA	71.0 (56–84)	69.9 (55–79)	0.68
At cement insertion	74.9 (61–85)	73.5 (60–87)	0.61
Diagnosis			0.27
Infected TKA	11	14	
Repeated infection after R-TKA	3	1	
Pathogen			
*MSSA*	3 (21.4%)	4 (26.7%)	
*Escherichia coli*	3 (21.4%)	3 (20.0%)	
*Staphylococcus agalactiae*	2 (14.3%)	1 (6.7%)	
*MRSA*	2 (14.3%)	2 (13.3%)	
*Staphylococcus epidermidis*	0	2 (13.3%)	
No growth	4 (28.6%)	3 (20.0%)	
Follow-up period, months	20.7 (14.1–33.2)	30.8 (13.8–54.0)	0.02

Data are presented as n (%) or mean (range)

TKA: total knee arthroplasty; R: revision; MSSA: methicillin-sensitive *Staphylococcus aureus*; MRSA: methicillin-resistant *Staphylococcus aureus*

### First-stage procedure

**Fig. 1:**
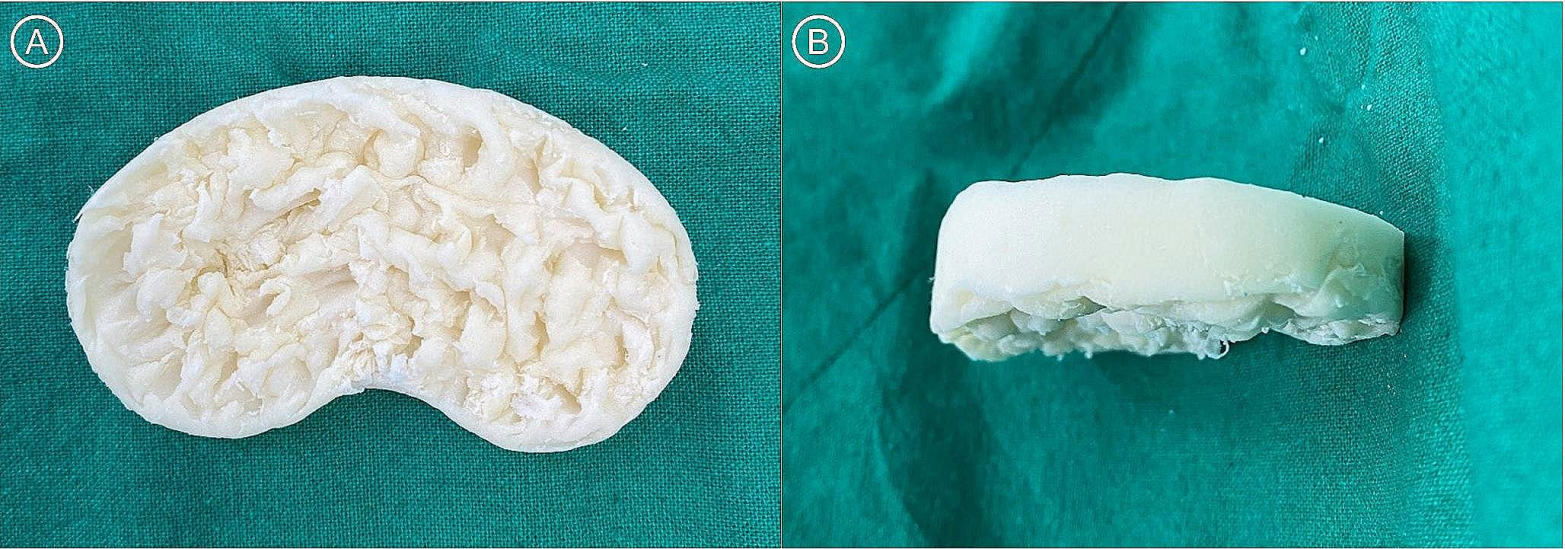
Photograph of a spiked tibial cement spacer. (**A**) Bottom surface. (**B**) Lateral view

A midline skin incision was made using an existing operative scar, a medial parapatellar approach was performed to open the knee joint, and a bacterial culture test was performed on the joint fluid. A meticulous total synovectomy was performed, the existing prosthesis was removed, and massive pulsatile irrigation was performed on the entire knee joint, including the intramedullary canal. In the conventional group, tibial and femoral cement spacers were made using a mold for ACS production. In the spiked TCS group, several spikes were made on the bottom of the TCS in the same manner as previously reported (Fig. 1) [12]. A negative drain was then inserted, and the wound was closed.

Follow-up revealed no inflammatory symptoms such as swelling, local heat, or tenderness, and normal CRP levels. The joint aspiration culture test was negative, and intravenous antibiotic treatment was discontinued. Following at least four weeks of intravenous antibiotics, the patients underwent an antibiotic-free period of at least two weeks to allow any residual infection to reappear. After discontinuation of antibiotics, blood tests and aspiration culture tests confirmed no abnormal findings and second-stage surgery was planned.

### Second-stage procedure

**Fig. 2:**
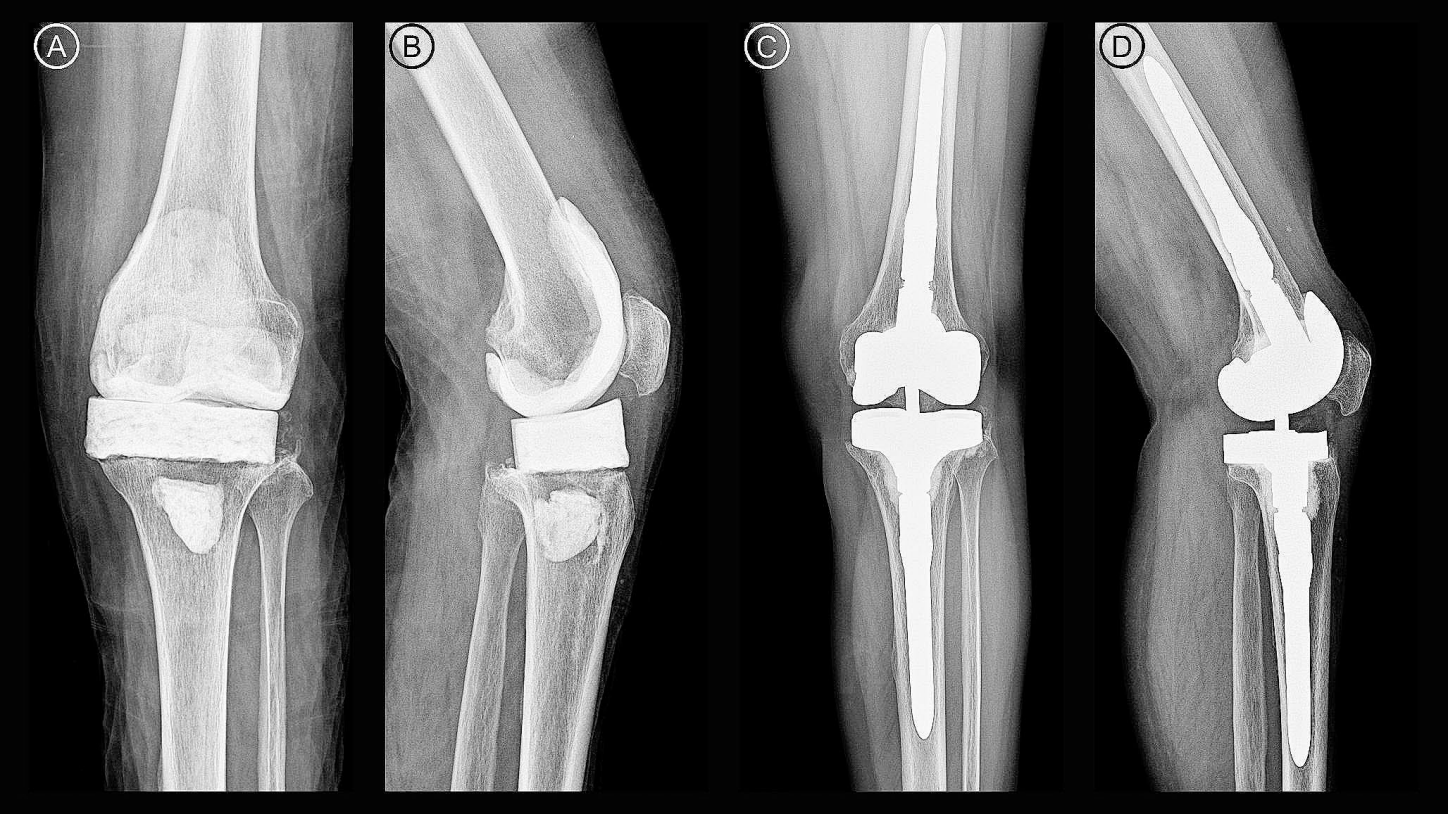
(**A**) Anteroposterior and (**B**) lateral radiographs obtained at first-stage revision arthroplasty using a spiked tibial cement spacer (**C**) anteroposterior and (**D**) lateral radiographs obtained at 21 months after second-stage revision arthroplasty using a revision system

A medial parapatellar arthrotomy was performed and intraoperative culture was performed on the joint fluid, but unexpected positive cultures were not identified in all patients [13]. Intraoperative frozen section examination revealed less than five WBCs per high-power field in all patients, the second stage procedure was performed as planned. All patients underwent R-TKA with the Triathlon^®^ revision system (Stryker, Kalamazoo, MI, USA) (Fig. 2).

### Clinical assessment

At the last follow-up, we assessed the functional outcomes of motion arc, numerical rating scale (NRS), and Knee Society (KS) scores. Additionally, to evaluate infection eradication, serum ESR and CRP levels, the number of cement spacer insertion repetitions, and recurrence of infection after R-TKA were examined.

### Statistical analysis

An independent Student’s t-test was used to compare the two groups. All statistical analyses were performed using SPSS Statistics for Windows (version 24, IBM Corp., Armonk, NY, USA). A *P* value less than or equal to 0.05 was considered statistically significant.

### Ethics approval

This study was approved by the Institutional Review Board (IRB) of our institution (IRB No. 2024-01-002) and was performed in accordance with the principles of the Declaration of Helsinki. Written informed consent was obtained from all the participants.

## Results

### Demographics

Table 1 presents the clinical characteristics of 29 patients who underwent two-stage revision arthroplasty. No significant differences were observed in the female ratio and mean age between the two groups. The mean number of days between first- and second-stage procedures was 55.9 days (range, 42–67) in Group S and 69.6 days (range, 49–123) in Group C, and the number of days between surgeries was significantly shorter in Group S than in Group C (*P* = 0.03). The mean follow-up period after R-TKA in Group S was 20.7 months, which was significantly shorter than the 30.8 months in Group S (*P* = 0.02).

### Functional outcomes

Table 2 presents the functional outcomes in the two groups after R-TKA. The mean motion arc was significantly larger in Group S (105.7°) than in Group C (88.7°, *P* = 0.002). The mean NRS was significantly lower in Group S (2.8) than in Group C (5.0, *P* = 0.002). The mean KS score in Group S (71.6) was significantly higher than that in Group C (48.2, *P* < 0.001).

**Table 2:** Comparison of motion arc, NRS, and KS score between the two groups

	Group S (*n* = 14)	Group C (*n* = 15)	*P* value
Motion arc, °	105 (90–120)	88.7 (60–110)	0.002
NRS	2.8 (0–6)	5.0 (2–8)	0.002
KS score	71.6 (54.3–88.4)	48.2 (27.4–78.7)	< 0.001

Data are presented as mean (range) or n (%)

NRS: numerical rating scale; KS: Knee Society

### Infection eradication

Table 3 presents the infection control parameters after R-TKA in the two groups. At the last follow-up, the mean serum ESR levels were 23.4 mm/h (range, 5–68 mm/h) and 35.5 mm/h (range, 2–103 mm/h) in Group S and C, respectively, with no significant difference between the two groups (*P* = 0.22). The mean serum CRP levels were 0.2 mg/dL (range, 0.1–0.51 mg/dL) in Group S and 0.5 mg/dL (range, 0.02–1.8 mg/dL) in Group C, with no significant difference between the two groups (*P* = 0.11). The number of cement spacer insertion repetitions was lower in Group S (0%) than in Group C (26.7%, *P* = 0.04). However, the recurrence rate after R-TKA was 14.3% and 13.3% in Groups S and C, respectively (*P* = 0.94).

**Table 3:** Comparison of erythrocyte sedimentation rate, C-reactive protein, and the rate of repeating the first-stage procedure and recurrence after revision total knee arthroplasty

	Group S (*n* = 14)	Group C (*n* = 15)	*P* value
ESR, mm/hr	23.4 (5–68)	35.5 (2–103)	0.22
CRP, mg/dL	0.19 (0.1–0.51)	0.47 (0.02–1.8)	0.11
Repeating the first stage surgery	0 (0%)	4 (26.7%)	0.04
Recurrence after R-TKA	2 (14.3%)	2 (13.3%)	0.94

Data are presented as mean (range) or n (%)

ESR: erythrocyte sedimentation rate; CRP: C-reactive protein, R-TKA: revision total knee arthroplasty

## Discussion

The results of our retrospective trial revealed that in two-stage revision arthroplasty for infected TKA or infected R-TKA, the group using spiked TCS had better functional results and a lower rate of first-stage re-operation than the group using conventional TCS. However, no significant difference in the recurrence rate was observed after R-TKA.

No significant difference was observed in demographics between the two groups; however, the mean follow-up period in Group S was significantly shorter than that in Group C. This is because the surgeon transitioned from conventional TCS to spiked TCS. Owing to the retrospective study design, the mean follow-up period of the two groups varies depending on the surgeon’s surgical technique. This may influence the interpretation of the results.

### Interval between the first-stage and revision arthroplasty procedures

Akhtar et al. [10] evaluated the mean interval between stages according to the type of cement used in two-stage revision arthroplasty. They reported an average of 275 days in the SCS group, 291 days in the conventional ACS group, and 233 days in the ACS with pedestal group, but no significant difference. Kim et al. [11] performed a two-stage revision using a modified ACS in infected TKA and reported the mean period between the first-stage procedure and revision arthroplasty to be 3.3 months (range, 3 to 4 months). In this study, the mean interval between stages was 55.9 and 69.6 days for Groups S and C, respectively, which was shorter than that reported in previous studies [10, 11]. Revision arthroplasty was performed within two months in Group S, which may help increase patient compliance.

### Motion arc after R-TKA

A retrospective study that compared five year outcomes between ACSs and static cement spacers (SCSs) reported that ACS resulted in significantly improved motion arc (111° vs. 82°, *P* < 0.001) [14]. A randomized trial of ACSs and SCSs reported a mean motion arc of 113.0° in the ACS group compared with 100.2° in the SCS group (*P* = 0.001) [15]. Systematic reviews have reported that ACS resulted in significantly greater motion arc after reimplantation [16, 17]. ACS has the potential for improved mobility and function by safely providing a more “livable” knee during the convalescent period before the final R-TKA [14].

### Functional outcomes following R-TKA

A retrospective study that compared five year outcomes between ACSs and SCSs reported that ACS resulted in significantly improved Short Form 12 physical component score (35.2 vs. 21.0, *P* = 0.01), KS score (145.2 vs. 113.7, *P* < 0.001), and Western Ontario and McMaster Universities Osteoarthritis Index functional scores (60.1 vs. 51.1, *P* = 0.03) compared to the SCS group [14]. A randomized trial of ACSs and SCSs reported that the mean KS score at a mean of 3.5 years was higher in the ACS group (79.4 vs. 69.8 points, *P* = 0.043) [15]. However, systematic reviews have reported similar functional scores in the treatment groups despite significant differences in ultimate knee motion [16, 17]. Lanting et al. [18] examined the outcome following subluxation of ACSs in two-stage R-TKA and reported a lower mean KS function score of 39.3 in patients with sagittal subluxation outside one standard deviation from the mean than in patients with sagittal subluxation within one standard deviation from the mean (60.2, *P* = 0.045). A previous study that evaluated the initial radiological stability of spiked TCS [12] reported that the degree of mediolateral translation of spiked TCS was significantly smaller than that of conventional TCS; therefore, we infer that the same initial stability would have been secured in this study. This initial stability, i.e. less sagittal subluxation, may have resulted in a significant improvement in functional scores following R-TKA.

### Number of cement spacer insertion repetitions before R-TKA

Akhtar et al. [10] evaluated the repetition of cement spacer insertion according to the type of cement used in two-stage revision arthroplasty. They discovered a total of two, two, and one repetition of cement spacer insertion in the SCS group, conventional ACS group, and ACS with pedestal group, respectively; however, no significant differences were reported. They interpreted that the number of cases with complications, including repetitions of cement spacer insertion, was relatively lower in the ACS with pedestal group than in the SCS or conventional ACS group because the positioning and balancing of the cement spacer were optimized. In this study, cement spacer insertion was repeated in four patients (26.7%) in Group C. In all cases, the reason for repeat cement spacer insertion was mechanical instability of the previously inserted cement spacer, such as subluxation or dislocation of TCS, rather than poor infection control. No cement spacer insertion was repeated in Group S. The rate of repetition of cement spacer insertion was low in the spiked TCS group because the initial mechanical stability of the spacer was better with spiked TCS than with conventional TCS, as observed in a previous study [12].

### Recurrence of infection following R-TKA

A case series study of ACSs in two-stage R-TKA reported no infection recurrence at a mean duration of 64.2 months [19]. Hoshino et al. [20] performed two-stage revision arthroplasty using ACS made using a handmade silicone mold and reported no recurrence of infection during a mean follow-up period of 54 months. A retrospective study that compared five year outcomes between ACSs and SCSs reported that infection eradication was similar in both groups (83.7% and 86.1%, respectively, *P* = 0.234) [14]. Some systematic reviews have reported similar reinfection rates between the two types of spacers [16, 17]. However, a systematic review compared the infection eradication rate after articulating versus static spacers in two-stage revision arthroplasty and reported that ACSs are associated with a lower recurrence of infection than SCSs at a comparable mean duration of follow-up [21].

### Limitations

This study has other limitations. First, although alpha-defensin and leukocyte esterase have been introduced as diagnostic markers for the accurate diagnosis of periprosthetic joint infection (PJI) [22], the authors of this study diagnosed PJI using only haematological tests, radiological tests, and bacterial culture tests. Second, the necessity of the extensile approach during R-TKA was not evaluated. However, R-TKA was performed without additional procedures, such as tibial tuberosity osteotomy, in all the 29 cases included in this study. Additional analysis was needed to determine whether complications such as poor wound recovery due to excessive soft tissue traction or prolongation of surgical time due to narrow field of view that may arise from using only the standard medial parapatellar approach without performing an extensile approach. Intraoperative findings such as extension/flexion gap balance and degree of bone defect during R-TKA were not assessed. In R-TKA, this limitation may be overcome through research that evaluates intraoperative soft tissue tension using equipment such as tensor/balancer device and torque driver [23]. However, more constrained types of implants, such as rotating hinge prostheses or constrained condylar knee prostheses, were not used in all cases [24]. Therefore, it can be assumed that the extension/flexion gap or mediolateral gap balancing was not poor [25].

## Conclusions

Compared with conventional TCS, spiked TCS shortened the period until R-TKA and improved pain and function levels in patients undergoing two-stage revision for infected TKA. However, no significant difference in the rate of infection recurrence was observed after R-TKA.

## Data Availability

The datasets used and/or analyzed during the current study are available from the corresponding author on reasonable request.
